# Effects of the Dietary Probiotic, *Enterococcus faecium* NCIMB11181, on the Intestinal Barrier and System Immune Status in *Escherichia coli* O78-Challenged Broiler Chickens

**DOI:** 10.1007/s12602-018-9434-7

**Published:** 2018-06-12

**Authors:** Liqing Huang, Liping Luo, Yaru Zhang, Zhong Wang, Zhaofei Xia

**Affiliations:** 1 College of Veterinary Medicine, China Agricultural University, Beijing, 100193 People’s Republic of China; 2 State Key Laboratory of Animal Nutrition, College of Animal Science and Technology, China Agricultural University, Beijing, 100193 People’s Republic of China

**Keywords:** Broilers, *Enterococcus faecium*, *Escherichia coli* O78, Immune response, Intestinal barrier function

## Abstract

The effects of *Enterococcus faecium* on growth, intestinal barrier function, and immune response in *Escherichia coli* O78-challenged broiler chickens were investigated. Three hundred eight 1-day-old Ross male chickens were randomly assigned into three treatment groups: negative control (C), *E. coli* O78-infected positive (EP), and *E. coli* O78-infected with 200 mg/kg *E. faecium* dietary supplementation (EF). *E. faecium* significantly increased the body weight on day 10 (*P* < 0.05) and day 15. Furthermore, these birds had a greater average daily gain compared with the other groups during days 1–10 (*P* < 0.05). The death rate of the EF chickens dramatically declined. *E. faecium* supplementation improved the jejunal villus height and the ratio of villus height to crypt depth (*P* < 0.05) 3 and 7 days post-infection. The mRNA expression of *claudin-1* significantly increased by *E. faecium* (*P* < 0.05) 3 and 7 days post-infection, and *Mucin2* was markedly enhanced (*P* < 0.05) 3 days post-infection. *E. faecium* upregulated the mRNA expression of *PPAR-γ* and *IL-10* (*P* < 0.05) and downregulated that of *NF-κB*, *TLR4*, and *IL-1β* (*P* < 0.05) in the spleen 3 and 7 days post-infection. Lipopolysaccharide stimulation index was markedly enhanced in the EF group (*P* < 0.05) 3 days post-infection. The increased liver *E. coli* number caused by the *E. coli* O78 challenge was significantly reversed by *E. faecium* (*P* < 0.05). *E. faecium* improved growth and reduced the death rate by regulating the immune response and maintaining the intestinal integrity in *E. coli* O78-challenged broiler chickens.

## Introduction

Avian colibacillosis, caused by specific serotypes or opportunistically pathogenic *Escherichia coli*, is one of the crucial bacterial diseases of poultry [1, 2]. Young birds, in which the protective immune system is not fully developed, are more vulnerable. *E. coli* serotypes O78:K80, O1:K1, and O2:K1 are the most commonly found in domestic breeds with colibacillosis [3]. Although various antibiotics are typically used to prevent and control colibacillosis, cumulative reports have demonstrated that drug resistance of *E. coli* has increased owing to the spreading of resistance genes such as extended-spectrum beta-lactamases (ESBL) and/or plasmid-mediated Amp-C beta-lactamases (Amp-C) [4, 5]. Therefore, potential antibiotic alternatives to reduce antimicrobial drug usage in poultry production are urgently needed.

*Enterococcus faecium*, a lactic acid-producing Gram-positive bacterium found in the intestine of healthy animals and humans, is a probiotic that may be beneficial for animal health [6, 7]. *E. faecium* increases the concentration of organic acids and bacteriocins, which are important for the alimentary tract because of their nutritional benefits for enterocytes and their inhibitory effects on pathogens [8]. Previous studies have indicated that *E. faecium* improves the metabolism of macronutrients [7], promotes growth performance [6, 9], inhibits pathogen proliferation [1, 10], improves intestinal morphology [6, 11], and enhances the immune response [12]. Additionally, *E. faecium* prevents *E. coli*-induced intestinal disorders and manipulates the cecal microflora [1]. *E. faecium* can also elicit protective immune responses by inducing cytokines, and T and B lymphocytes against *Salmonella* spp. [8, 13]. However, there are limited published reports on the effects of *E. faecium* on *E. coli*-challenged broiler chickens. Hence, the present study was designed to investigate the effects of *E. faecium* on growth performance, intestinal barrier function, and the innate immune response in broilers challenged with *E. coli* O78.

## Methods

### Experimental Design

The experimental animal protocol in this study was approved by the Animal Care and Use Committee of China Agricultural University (permit number 20121209–1). A total of 216 1-day-old birds were randomly assigned into three groups with six replicates of each. Each replicate consisted of 12 birds. Treatments were set as follows: negative control birds were fed a basal diet and injected with sterile saline (0.2 ml) in the left thoracic air sac (C); positive control birds were fed a basal diet and challenged at 11 days of age with *E. coli* O78 [0.2 ml, 10^4^ colony-forming unit (CFU)/ml] injected into the left thoracic air sac (EP) [2]; probiotic birds were fed a basal diet containing *E. faecium* and challenged with 0.2 ml, 10^4^ CFU/ml *E. coli* O78 that was injected into the left thoracic air sac (EF). The basal diet formula met or exceeded the nutrient requirements for broiler chickens recommended by the National Research Council (1994) and the diet composition is listed in Table 1. The total feeding period was 20 days.

**Table 1:** Composition and nutrient level of diet

Items (%)	Content
Ingredient	
Corn	56
Wheat	2
Soybean meal	34.5
Soybean oil	3.2
Dicalcium phosphate	1.85
Limestone	1.2
DL-Met	0.2
l-Lys-HCl	0.25
Sodium chloride	0.3
Choline chloride (50%)	0.2
Santoquin	0.05
Maifanite	0.03
Vitamin premix^a^	0.02
Trace mineral premix^b^	0.2
Nutrient and energy level	
ME (Mcal/kg)	2.96
Protein	19.98
Calcium	0.97
Lysine	1.19
Methionine	0.51
Available phosphorus	0.43

^a^The vitamin premix supplied the following (per kg of diet): vitamin A, 12500 IU; vitamin D_3_, 2500 IU; vitamin E 18.75 IU; vitamin K_3_, 2.65 mg; vitamin B_1_, 2 mg; vitamin B_2_, 6 mg; vitamin B_6_, 6 mg; vitamin B_12_, 0.025 mg; d-biotin, 0.0325 mg; folic acid, 1.25 mg; d-calcium pantothenate, 12 mg; nicotinic acid 50 mg

^b^The trace mineral premix supplied the following (per kg of diet): copper, 8 mg; zinc,75 mg; iron, 80 mg; manganese,100 mg; selenium, 0.15 mg; iodine,0.35 mg

#### *E. faecium* NCIMB11181 Preparation

The *E. faecium* NCIMB11181 preparation used in this study was a commercial product purchased from Probiotics International Ltd. (Stoke Sub Hamdon, Somerset, UK), which contained a total bacteria count ≥ 2.00 × 10^12^ CFU/kg. Sample testing showed that the bacteria count was 8.2 × 10^12^ CFU/kg. The *E. faecium* product (200 mg/kg) was carefully mixed into the basal diet. The diets in this experiment were manufactured at the feed mill of China Agricultural University. Finally, the actual diet contained 5.1 × 10^10^ CFU/kg *E. faecium*.

#### *E. coli* O78 Preparation

The *E. coli* O78 (CVCC1555) used in this study was purchased from China General Microbiological Culture Collection Center, Beijing, China. The strain was aerobically incubated in Luria-Bacterial liquid medium for 24 h at 37 °C with shaking (120 rpm). Before the challenge, the bacteria were centrifuged at 2800×*g* for 10 min and washed three times with phosphate-buffered saline (PBS). The bacterial concentration was measured with a spectrometer at 600 nm. PBS was used to adjust the suspension to the desired bacterial concentration.

#### Sample Collection

On days 15 and 20, one bird of each replicate was randomly selected and euthanized by sodium pentobarbital (30 mg/kg). The small intestine was removed and gently flushed with PBS. Jejunum samples were collected for intestinal morphology, and mucosal antibody and mRNA expression. Liver samples for bacterial count and spleen samples for mRNA expression were taken. All samples for mRNA expression were immediately frozen in liquid nitrogen.

### Growth Performance

Body weight (BW) for each replicate was measured on d1, d10 (the day before the challenge), d15 (3 days post-infection), and d20 (7 days post-infection). The average daily gain (ADG) was calculated during d1–10, d10–15, and d10–20. The death rate was calculated during d1–10 and d10–20.

### Intestinal Morphology

Jejunum segments were fixed with 4% paraformaldehyde and embedded in paraffin after 48 h. For morphological examination, tissue sections (5 μm) were stained with hematoxylin and eosin. Nine complete villi were measured in each section. The villus height was measured from the tip of the villus to the villus-crypt junction, and crypt depth was measured from the bottom of the villus to the lamina propria. Then, the villus height/crypt depth ratio was calculated. All the observations and measurements were performed with an Olympus optical microscope and ProgRes CapturePro software (version 2.7; Jenoptik, Jena, Germany).

### Mucosal IgA

Mucosal scraping was collected with sterile slides from two 5-cm jejunal samples and homogenized in saline. The secretory IgA (sIgA) concentration was measured by an ELISA kit (Bethyl Laboratories Inc., Montgomery, TX, USA) and the total protein of the mucosal homogenate was determined by a BCA protein assay kit (Thermo Fisher Scientific, Waltham, MA, USA) according to the manufacturers’ protocols. The sIgA concentration is presented as milligrams per gram of protein.

### Liver Bacterial Count

Two hundred micrograms of liver tissue per sample was placed in 0.2 ml sterile saline and homogenized by a stomacher. Each homogenate was diluted from 10^−1^ to 10^−6^ with sterile saline. Each diluted sample (0.1 ml) was incubated on a MacConkey agar plate (Land Bridge Technology, Beijing, China) at 37 °C for 24 h. Plates containing 30–300 bacterial colonies were selected to count. The final number is shown as log_10_ (CFU/g tissue).

### Peripheral Blood Mononuclear Cell Isolation

Blood was collected into heparin anticoagulant tubes on d15 and d20. Peripheral blood mononuclear cells (PBMCs) were separated by Ficoll solution (Histopaque1077; Sigma-Aldrich co., St. Louis, MO, USA). Uncoagulated blood was diluted with Hanks solution at 1:1 (no calcium and magnesium, Thermo Fisher Scientific) and layered on top of the Ficoll solution in a 10-ml centrifuge tube (2:1). After centrifugation for 30 min at 1000×*g* (20 °C), the PBMCs at the plasma-Ficoll interface were collected carefully. Then, cold RPMI-1640 medium (containing 5.0% inactivated fetal bovine serum, 0.0599 mg/ml penicillin, 100 μg/ml streptomycin, and 24 mM HEPES) was used to wash the PBMCs three times with centrifugation at 250×*g* for 10 min (4 °C). The PBMC count was evaluated by trypan blue staining.

### Flow Cytometry for Lymphocyte Subpopulation Analysis

Peripheral lymphocytes contained in PBMC fraction as prepared above were stained with chicken CD3 (SPRD, clone: CT-3), CD4 (FITC, clone: CT-4), and CD8 (PE, clone: CT-8), and incubated in a water bath for 30 min. All the antibodies used in the study were purchased from SouthernBiotech (Birmingham, AL, USA). Then, lymphocytes were washed twice with Hanks solution and fixed with 3% paraformaldehyde solution. The analysis was conducted by a multi-channel cytometer (Beckman-Coulter, Carlsbad, CA, USA). The results are presented as the percentage of positive lymphocyte subpopulation with the specific antibody.

### Lymphocyte Proliferative Responses

The method for PBMC isolation was described above. Trypan blue staining was used to evaluate cell count and viability. The proliferative response of T and B lymphocytes was measured by the MTT assay, after stimulation with Concanavalin A (Con A; from *Canavalia ensiformis*; Sigma-Aldrich) and lipopolysaccharides (LPS; from *E. coli*; Sigma-Aldrich), respectively. Results are expressed as stimulation index (SI).

### Total RNA Extraction and Real-time Quantitative PCR

Total RNA of spleen and jejunal tissues was extracted by TRIzol reagent (Invitrogen, Carlsbad, CA, USA) following the manufacturer’s protocol. The RNA concentration was measured by a nanodrop spectrophotometer (ND-2000, Thermo Fisher Scientific) at 260 and 280 nm. The total RNA purity was verified by the 260 nm/280 nm ratio; the results of all samples were between 1.8 and 2.0. Then, 2 μg of total RNA was used for reverse transcription by a commercial kit (Takara Biotechnology Co. Ltd., Tokyo, Japan) following the manufacturer’s protocol. The complementary DNA was stored at − 20 °C.

The expression of inflammation-related genes and tight junction (TJ) genes in the spleen and jejunum were determined by real-time quantitative PCR (RT-PCR). Gene primer sequences are presented in Table 2. RT-PCR was performed on an Applied Biosystems 7500 Fast Real-time PCR System (Applied Biosystems, Carlsbad, CA, USA) using a commercial SYBR Green kit (Takara Biotechnology Co. Ltd.). According to the manufacturer’s protocol, the initial denaturation phase was set at 95 °C for 5 min followed by 40 cycles of 95 °C for 30 s and 60 °C for 30 s during annealing and extension. Melting curve analysis was used to evaluate the specificity of the amplified products. All the genes in this study were analyzed using *GAPDH* as an endogenous reference gene. The average gene expression level relative to *GAPDH* of each sample was calculated using the 2^−ΔΔCt^ method.

**Table 2:** Primers for real-time quantitative PCR assay

Gene	Primer sequence (5′–3′)	Accession no.
Mucin-2	F: TTCATGATGCCTGCTCTTGTG	XM_421035
	R: CCTGAGCCTTGGTACATTCTTGT	
ZO-1	F: CTTCAGGTGTTTCTCTTCCTCCTC	XM_413773
	R: CTGTGGTTTCATGGCTGGATC	
Claudin-1	F: CATACTCCTGGGTCTGGTTGGT	AY750897.1
	R: GACAGCCATCCGCATCTTCT	
Occludin	F: ACGGCAGCACCTACCTCAA	D21837.1
	R: GGGCGAAGAAGCAGATGAG	
NF-κb	F: GTGTGAAGAAACGGGAACTG	NM205129
	R: GGCACGGTTGTCATAGATGG	
PPARγ	F: GACCTTAATTGTCGCATCCAT	AF163811
	R: CGGGAAGGACTTTATGTATGA	
TLR4	F: AGTCTGAAATTGCTGAGCTCAAAT	NM_001030693
	R: GCGACGTTAAGCCATGGAAG	
TNF-α	F: GAGCGTTGACTTGGCTGTC	NM_204267
	R: AAGCAACAACCAGCTATGCAC	
IL-1β	F: ACTGGGCATCAAGGGCTA	NM_204524
	R: GGTAGAAGATGAAGCGGGTC	
IL-6	F: TTTATG GAGAAGACCGTGAGG	NM_204628
	R: TGTGGCAGATTGGTAACAGAG	
IL-10	F: GCTGTCACCGCTTCTTCACCT	EF554720.1
	R: GGCTCACTTCCTCCTCCTCATC	

*F*, forward; *R*, reverse

### Statistical Analysis

Data were analyzed by one-way ANOVA using SPSS 17.0 software (version 17.0, SPSS Inc., Chicago, IL, USA). Statistical differences among treatments were examined by Duncan’s multiple range test. Results are presented as the mean ± SE. Differences were considered statistically significant at *P* < 0.05, and 0.05 < *P* < 0.1 was regarded as a trend towards significance.

## Results

### Growth Performance

**Fig. 1:**
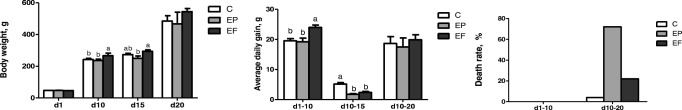
Effects of dietary *Enterococcus faecium* on growth performance (body weight, average daily gain, and death rate) of broilers. C, birds fed with basal diet; EP, birds fed with basal diet and challenged with *E. coli* O78; EF = birds fed a basal diet supplemented with *E. faecium* and challenged with *E. coli* O78. Bars with letters (a–c) suggested significant difference among different treatments (*P* < 0.05)

The growth performance parameters are shown in Fig. 1. Compared with the control group, *E. faecium* supplementation significantly increased the BW of chickens on d10 (*P* < 0.05) right before the *E. coli* challenge, and on d15 (3 days after the *E. coli* challenge). The EF birds had a greater ADG than the other birds during d1–10 (*P* < 0.05). The death rate was dramatically lower in the EF group compared with that in the EP group. However, the *E. coli* challenge markedly decreased the BW on d15 (*P* < 0.05) and the ADG during d10–15 (*P* < 0.05). The death rate of the EP group was the highest among all three groups. No significant differences were observed in BW on d1 and 20, and in the ADG from d10 to d20.

### Intestinal Morphology

**Fig. 2:**
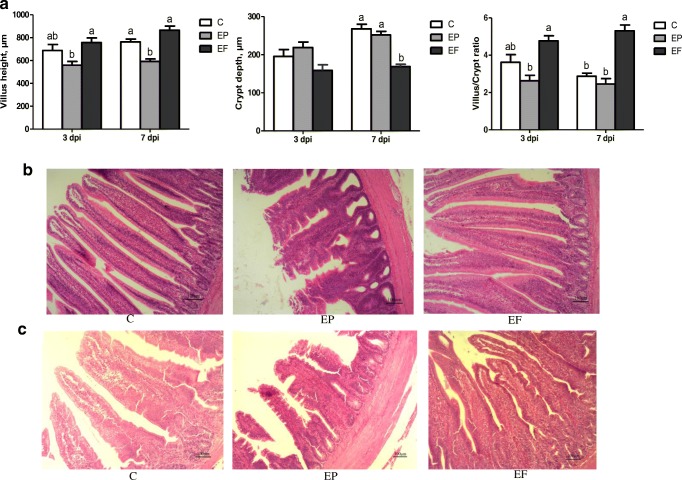
Effects of *Enterococcus faecium* on the jejunum morphology of broilers. (A) Statistic data of villus height, crypt depth, and villus/crypt ratio (V/C). (B) and (C) Photomicrographs of jejunum contained from C, EP, and EF group, on day 3 and 7 post-infection, respectively (× 100). C, birds fed with basal diet; EP, birds fed with basal diet and challenged with *E. coli* O78; EF, birds fed a basal diet supplemented with *E. faecium* and challenged with *E. coli* O78. Bars with small letters (a–c) suggested significant difference among different treatments (*P* < 0.05)

**Fig. 3:**
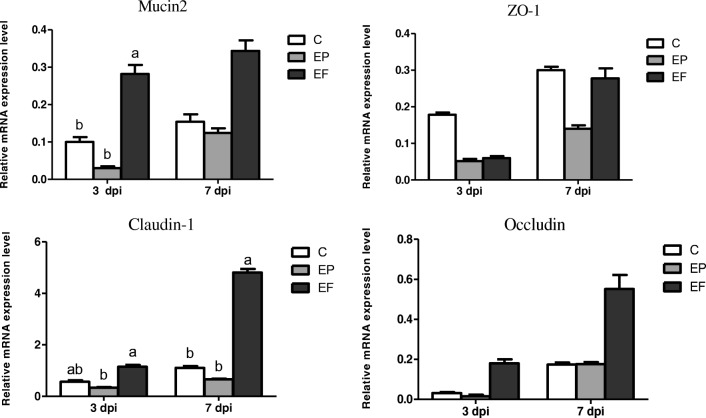
The expression of intestinal tight junction proteins and *mucin2* gene in the jejunum. C, birds fed with basal diet; EP, birds fed with basal diet and challenged with *E. coli* O78; EF = birds fed a basal diet supplemented with *E. faecium* and challenged with *E. coli* O78. Bars with letters (a–c) suggested significant difference among different treatments (*P* < 0.05)

According to Fig. 2(A), 3 and 7 days post-infection, the *E. coli* challenge significantly decreased the jejunal villus height (*P* < 0.05), and the birds in the EF group had a markedly higher jejunal villus height than those in the other groups (*P* < 0.05). The *E. coli* infection significantly increased the crypt depth 3 and 7 days post-infection (*P* < 0.05). *E. faecium* markedly increased the villus/crypt ratio (*P* < 0.05) 3 and 7 days post-infection. Histopathological changes (Fig. 2(B, C)) showed that the *E.coli* infection caused shedding and swelling of villus tip and increased crypt depth 3 and 7 days post-infection. The supplementation of *E. faecium* maintained intact structural of jejunum and increased villus height 3 and 7 days post-infection.

### Relative mRNA Expression of TJ Proteins and *Mucin*

The relative mRNA expression of different intestinal TJ proteins (zonula occludens-1, claudin-1, and occludin) and the *Mucin2* gene is shown in Fig. 3. *Mucin2* mRNA expression in the jejunum significantly decreased after *E. coli* infection (*P* < 0.05). However, the EF group showed a significant increase in *Mucin2* mRNA 3 days post-infection (*P* < 0.05) and an increased tendency 7 days post-infection (*P* = 0.083). The *E. coli* infection and the *E. faecium* supplementation did not affect the zonola occludens-1 (*ZO-1*) mRNA expression 3 and 7 days post-infection (*P* > 0.05). *E. faecium* addition markedly increased *claudin-1* mRNA expression 3 and 7 days post-infection (*P* < 0.05). The *E. faecium* supplementation also tended to upregulate *occludin* mRNA expression 3 and 7 days post-infection (*P* = 0.053 and *P* = 0.051, respectively).

### Evaluation of Cellular and Humoral Immunity

The peripheral blood lymphocyte phenotypes of the different treatments are summarized in Table 3. No remarkable differences were observed in the percentage of CD3+, CD4+, and CD8+ cells, as well as the ratio of CD4+/CD8+ among the three treatments 3 and 7 days post-infection (*P* > 0.05).

**Table 3:** Phenotyping of lymphocytes in peripheral blood of broilers in different treatments

Items	Treatments			SEM	*P* value
	C	EP	EF		
3 dpi					
CD3+	52.1 ± 5.69	59.58 ± 2.96	53.53 ± 4.28	2.521	0.481
CD4+	31.78 ± 3.38	36.5 ± 2.11	34.6 ± 2.81	1.582	0.515
CD8+	19.28 ± 2.34	20.43 ± 0.82	17.13 ± 2.05	1.052	0.472
CD4+/CD8+	1.66 ± 0.06	1.79 ± 0.11	2.16 ± 0.41	0.145	0.375
7 dpi					
CD3+	86.43 ± 2.16	84.35 ± 2.58	87.38 ± 0.96	1.122	0.577
CD4+	60.1 ± 1.38	55.9 ± 1.79	59.38 ± 1.42	0.976	0.176
CD8+	25.58 ± 1.49	27.05 ± 1.43	29.33 ± 0.3	0.784	0.142
CD4+/CD8+	2.38 ± 0.15	2.08 ± 0.08	2.03 ± 0.06	0.073	0.094

*C*, birds fed with basal diet; *EP*, birds fed with basal diet and challenged with *E. coli* O78; *EF*, birds fed a basal diet supplemented with *E. faecium* and challenged with *E. coli* O78

The function of peripheral blood lymphocytes among the three treatments 3 days post-infection was tested in vitro (Table 4). The proliferative response of T lymphocytes was not affected by *E. coli* infection or *E. faecium* supplementation (*P* > 0.05), as evidenced by ConA SI. However, *E. faecium* significantly increased the LPS SI (*P* < 0.05), which reflects the active response of B lymphocyte proliferation in the EF group.

**Table 4:** The function of peripheral blood lymphocytes of broilers in different treatments on day 3 post-infection

Items	Treatments			SEM	*P* value
	C	EP	EF		
ConA SI	0.7 ± 0.070	0.66 ± 0.020	0.78 ± 0.040	0.030	0.212
LPS SI	0.73 ± 0.05^b^	0.68 ± 0.02^b^	1.15 ± 0.14^a^	0.088	0.023

^a, b^Means in the same row, values with different small letter superscripts, show significant difference (*P* < 0.05)

*C*, birds fed with basal diet; *EP*, birds fed with basal diet and challenged with *E. coli* O78; *EF*, birds fed a basal diet supplemented with *E. faecium* and challenged with *E. coli* O78

### Intestinal Immune Responses (sIgA)

The concentration of the jejunum sIgA was not influenced by either the *E. coli* challenge or the *E. faecium* supplementation 3 and 7 days post-infection (*P* > 0.05) (data not shown).

### Spleen Inflammation-Related Gene Expression

**Fig. 4:**
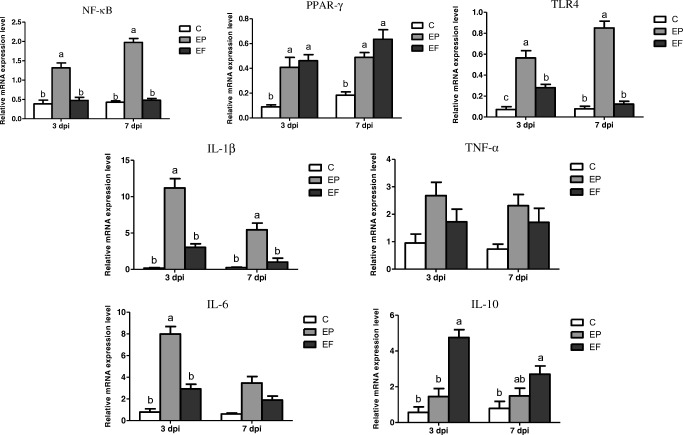
Relative mRNA expression levels of spleen inflammation-related cytokines of broilers in different treatments. C, birds fed with basal diet; EP, birds fed with basal diet and challenged with *E. coli* O78; EF, birds fed a basal diet supplemented with *E. faecium* and challenged with *E. coli* O78. Bars with letters (a–c) suggested significant difference among different treatments (*P* < 0.05)

The relative mRNA expression levels of spleen immune cytokines are presented in Fig. 4. Three days post-infection, the *E. coli* challenge significantly increased the expression of nuclear factor-κB (*NF-κB*), peroxisome proliferator-activated receptor*-γ* (*PPAR-γ*), toll-like receptor 4 (*TLR4*), *IL-1β*, and *IL-6* (*P* < 0.05) and tended to increase the expression of *TNF-α* (*P* = 0.052). The *E. faecium* supplementation markedly decreased the expression of *NF-κB*, *TLR4*, *IL-1β*, and *IL-6* (*P* < 0.05), and significantly enhanced the expression of *PPAR-γ* and *IL-10* (*P* < 0.05). Seven days post-infection, the EP birds had remarkably higher expression levels of *NF-κB*, *PPAR-γ*, *TLR4*, and *IL-1β* (*P* < 0.05). Furthermore, the *E. coli* infection tended to increase the expression of *TNF-α* and *IL-6* (*P* = 0.057 and *P* = 0.092, respectively). Dietary addition of *E. faecium* significantly elevated the expression of *NF-κB*, *PPAR-γ*, and *IL-10* (*P* < 0.05), and downregulated the expression of *TLR4* and *IL-1β* (*P* < 0.05). The birds in the EF group had a relatively lower *TNF-α* and *IL-6* expression (*P* = 0.057 and *P* = 0.092, respectively).

### Liver Bacterial Translocation

**Fig. 5:**
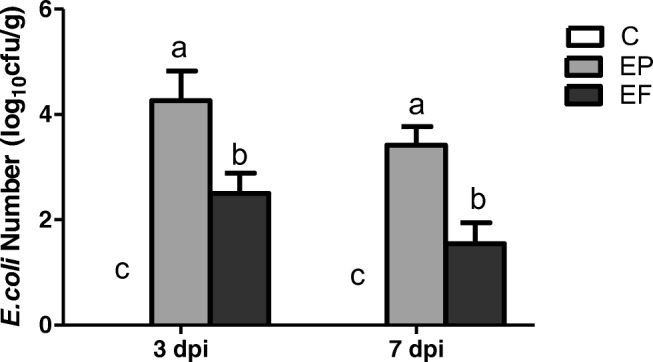
Liver bacterial translocation of *E. coli* in broilers (log_10_ cfu/g). C, birds fed with basal diet; EP, birds fed with basal diet and challenged with *E. coli* O78; EF, birds fed a basal diet supplemented with *E. faecium* and challenged with *E. coli* O78. Bars with letters (a–c) suggested significant difference among different treatments (*P* < 0.05)

As presented in Fig. 5, *E. coli* infection caused considerable liver bacterial translocation in broiler chickens. The *E. coli* challenge significantly increased the liver *E. coli* number (*P* < 0.05) 3 and 7 days post-infection. Compared with the EP group, the EF group had a markedly lower number of liver *E. coli* (*P* < 0.05) 3 and 7 days post-infection.

## Discussion

Probiotics have been proven to be beneficial for broiler breeding [14, 15]. *E. faecium* is a *Lactobacillus* genus that shows many positive effects on broiler growth and immunity [6, 7, 11, 12].

*E. coli* is a Gram-negative bacterium and its core pathogenic element is LPS [5, 16]. LPS can trigger system inflammation and cause death. Inflammation limits the synthesis of muscle protein and mobilizes energy to support the immune response, resulting in poor growth [16]. In the present study, chickens infected with *E. coli* O78 had a lower BW and ADG 3 days post-infection and the highest death rate during the entire feeding period. Many studies have shown that *E. faecium* can improve broiler performance [6, 9]. Cao et al. [1] have indicated that *E. faecium* enhanced chicken growth performance after an *E. coli* K88 challenge. Similarly, we showed that dietary *E. faecium* supplementation increased BW and ADG both before and 3 days post-infection and decreased the death rate.

Intestinal morphology reflects the health and integrity of the alimentary tract. A decreasing crypt size indicates the reconstruction of intestinal villus by accelerating the regenerative rate of enterocytes [14]. Therefore, the gut can resist devastating damage by pathogens or toxins. However, increased villus suggests the expansion of the intestinal absorption area, accumulation of mature enterocytes, and strengthening of the absorption and digestion ability [17]. Zhang et al. [5] have found that an *E. coli* K88 challenge disrupted intestinal morphology. Furthermore, *E. faecium* efficiently improved the intestinal mucosal architecture by increasing the villus height and decreasing the crypt depth [6]. According to Jin et al. [10], *E. faecium* can inhibit the adhesion of *E. coli* to enterocytes potentially through modifying the lumen pH and altering steric hindrance. Cao et al. [1] have reported that *E. faecium* was beneficial for the jejunal morphology of *E. coli* K88-challenged broilers compared with the control and antibiotic groups. Consistently, we showed that the addition of *E. faecium* improved the intestinal histomorphology with an increased V:C ratio and villus height in the challenged birds 3 and 7 days post-infection.

The intestinal mechanical barrier, which is composed of enterocytes and TJs, plays a key role in the natural defense against pathogen invasion [18, 19]. The major TJ proteins, including occludin, claudin and zonula occludens (ZO), and junctional adhesion molecule (JAM) [20], play an important role in maintaining intestinal permeability and the mucosal barrier function by sealing the extracellular space between the epithelial cells. In this study, the *E. coli* O78 challenge significantly reduced the expression of *claudin-1* 3 and 7 days post-infection, which is consistent with the findings of Gadde et al. [21] and Lee et al. [22], who showed that LPS treatment decreased the expression of TJ proteins. Probiotics have been proven to enhance TJ protein excretion and strengthen the mucosal barrier [23]. Feeding chickens with *E. faecium* upregulated the expression of *claudin-1* 3 and 7 days post-infection. The mucosal layer above the gastrointestinal tract is the chemical barrier of the intestine and mucins are the fundamental components [24]. Mucin-2, acting as a major *mucin* gene in the small intestine, was significantly increased 3 days post-infection by the *E. faecium* supplementation in the present study. This result is consistent with Gadde et al. [21], who revealed that probiotics increased the expression of the *Mucin2* gene in LPS-challenged chickens. These results suggest that *E. faecium* addition counteracted the detrimental effects of *E. coli*, and significantly enhanced *claudin-*1 and *Mucin2* expression, especially at the early infection stage. However, *E. faecium* did not affect the gene expression of other TJ proteins.

The spleen, a vital immune organ involved in both cellular and humoral immune responses, is important for lymphocyte generation, maturation, and storage [25]. Thus, immune-mediated spleen gene expression is considered to be an indicator of system immunity [26]. LPS is the primary cytoderm component of Gram-negative bacteria, and the LPS endotoxin serves as an activator of the innate immune response [27]. *E. coli* O78 can release LPS. Pattern recognition receptors (PRRs) have been selected to recognize the conserved elements of pathogens during the process of evolution [28]. TLRs are crucial members of PRRs and TLR4 can recognize LPS [29]. After binding to LPS, avian TLR4 triggers a cascade of inflammation responses via the myeloid differentiation factor 88 (MyD88)-dependent signaling pathway, which results in NF-κB activation [30]. NF-κB is the key factor in the regulation of the secretion of various cytokines and inflammatory mediators [31]. Many studies have used LPS injection to trigger a pro-inflammatory cytokine response in broiler chickens [16, 27, 31, 32], which resulted in activated TLR4. Similarly, in the current study, the infection of *E. coli* O78 significantly increased the expression of TLR4 and NF-κB 3 and 7 days post-infection. PPARs are key regulators of inflammatory and immune responses [16]. Cumulative research has proven that PPAR-γ ligands can inhibit major inflammation signaling pathways such as NF-κB, which implies the anti-inflammatory effect of PPAR-γ [33]. We found that *E. faecium* supplementation downregulated the expression of TLR4 and NF-κB, and upregulated PPAR-γ expression 3 and 7 days post-infection, which is consistent with the results by Gadde et al. [21].

Pro-inflammatory cytokines such as IL-1β, IL-6, and TNF-α regulate the immune response by inducing differentiation and proliferation of leukocytes to eliminate pathogens [34]. However, excessive secretion leads to organ damage and exacerbated energy consumption [34]. Thus, the suppression of IL-1β, IL-6, and TNF-α by dietary *E. faecium* supplementation in the current study may alleviate system inflammation. Conversely, Cao et al. [1] have indicated that *E. faecium* had no effect on the immune response to an *E. coli* K88 challenge. Different challenge approaches and measuring methods may be the reasons for this discrepancy. However, IL-10, which is a critical anti-inflammatory cytokine, acts as an inflammation feedback factor to modulate the immune response [35]. An LPS challenge suppressed the expression of anti-inflammatory cytokines [16, 21]. Our study found the same downregulation of IL-10 expression after *E. coli* O78 infection. Siepert et al. [12] have suggested that *E. faecium* supplementation elevated IL-10 expression. In the present study, addition of *E. feacium* markedly increased the IL-10 gene expression. Hence, we assume that *E. feacium* might regulate the NF-κB pathway by interacting with TLR4 and PPAR-γ, to alleviate the system inflammation caused by *E. coli* O78 infection.

Cellular and humoral immunity is the primary defense mechanism to obliterate pathogens [13]. CD4+ and CD8+ T cells play an important role in the cellular immune response. CD4+ T lymphocytes enhance the intercellular killing by macrophages and promote the expansion of cytotoxic T lymphocytes. CD8+ T lymphocytes are mainly involved in the elimination of antigens. CD3+ presents a surface marker of mature T lymphocytes [36]. The ratio of CD4+/CD8+ indicates the level of cell immunity [8]. The effect of *E. faecium* on animal immunity is controversial. Wang et al. [37] have demonstrated that the *E. faecium* supplementation elevated the activation of T helper lymphocytes and cytotoxic T lymphocytes in the peripheral blood of piglets infected with swine influenza. Levkut et al. [8] have indicated that *E. faecium* addition increased the number of CD3+, CD4+, and CD8+ cells in the peripheral blood of broiler chickens challenged with *Salmonella enteritidis*. In contrast, Kreuzer et al. [38] have found that *E. faecium* had no effect on the cellular immune response in lymph nodes and blood of piglets subjected with *Salmonella enterica*. In the current study, the differences in CD3+, CD4+, CD8+, and CD4+/CD8+ were not obvious among the three treatments, both 3 and 7 days post-infection. This result suggests that *E. faecium* may have a limited effect on immune mediation. Alternatively, it may be that cellular immunity has a minor effect on extracellular bacteria such as *E. coli*. At the early infection stage, the result of lymphocyte proliferative responses indicated that dietary *E. faecium* supplementation had no effect on T lymphocytes, as shown by ConA SI, whereas it significantly elevated B lymphocyte proliferation, as shown by LPS SI. Therefore, these results further indicate that the protective immune response evoked by *E. faecium* mainly focused on humoral immunity rather than cellular immunity. Interestingly, the *E. coli* O78 challenge reduced the LPS SI. According to Shini et al. [39], the endotoxins released by LPS induce degeneration and destruction of lymphocytes in birds. SIgA is a crucial immunoglobulin that serves as the first line of defense against pathogens [40]. Several studies have shown that probiotics can increase intestinal IgA excretion [40, 41]. However, the *E. faecium* supplementation did not result in overt upregulation of sIgA, both 3 and 7 days post-infection, which was similar to a previous study using the same challenging pathogen [1].

Bacterial translocation, defined as intestinal bacteria moving from the lumen to the mesentery or other parenteral organs, occurs frequently in damaged intestine [42]. Therefore, bacterial translocation is a useful indicator of the integrity of the intestinal structure [43]. In the present study, *E. faecium* supplementation markedly decreased the number of *E. coli* colonies in the liver of broiler chickens challenged with *E. coli* O78 3 and 7 days post-infection. The results manifested that *E. faecium* had the ability to maintain the intestinal integrity and limit the high permeability of the intestine caused by *E. coli* infection.

## Conclusions

Dietary *E. faecium* supplementation improved the growth performance and reduced the death rate of broiler chickens by enhancing the humoral immune response, modulating inflammatory cytokine secretion, enhancing TJ proteins’ expression, and maintaining the intestinal barrier against *E. coli* O78 infection. The beneficial effects of *E. faecium* may be partially associated with its effects on intestinal integrity and system humoral immunity. More studies are needed to further explore the potential mechanism of *E. faecium* in intestinal immunity.
